# Thermal and/or Microwave Treatment: Insight into the Preparation of Titania-Based Materials for CO_2_ Photoreduction to Green Chemicals

**DOI:** 10.3390/molecules29153646

**Published:** 2024-08-01

**Authors:** Iwona Pełech, Daniel Sibera, Piotr Staciwa, Konrad Sobczuk, Ewelina Kusiak-Nejman, Agnieszka Wanag, Antoni W. Morawski, Kenneth Schneider, Richard Blom, Urszula Narkiewicz

**Affiliations:** 1 Department of Inorganic Chemical Technology and Environment Engineering, Faculty of Chemical Technology and Engineering, West Pomeranian University of Technology in Szczecin, Pułaskiego 10, 70-322 Szczecin, Poland; daniel.sibera@zut.edu.pl (D.S.); piotr.staciwa@zut.edu.pl (P.S.); sk43128@zut.edu.pl (K.S.); ewelina.kusiak@zut.edu.pl (E.K.-N.); agnieszka.wanag@zut.edu.pl (A.W.); antoni.morawski@zut.edu.pl (A.W.M.); urszula.narkiewicz@zut.edu.pl (U.N.); 2 Department of Construction and Road Engineering, Faculty of Civil and Environmental Engineering, West Pomeranian University of Technology in Szczecin, Piastów 50a, 70-311 Szczecin, Poland; 3 Department of Process Technology, SINTEF Industry, Forskningsveien 1, 0373 Oslo, Norway; kenneth.schneider@sintef.no (K.S.); richard.blom@sintef.no (R.B.)

**Keywords:** titanium dioxide, thermal treatment, microwaves, carbon dioxide, photocatalysis, hydrogen

## Abstract

Titanium dioxide was synthesized via hydrolysis of titanium (IV) isopropoxide using a sol–gel method, under neutral or basic conditions, and heated in the microwave-assisted solvothermal reactor and/or high-temperature furnace. The phase composition of the prepared samples was determined using the X-ray diffraction method. The specific surface area and pore volumes were determined through low-temperature nitrogen adsorption/desorption studies. The photoactivity of the samples was tested through photocatalytic reduction of carbon dioxide. The composition of the gas phase was analyzed using gas chromatography, and hydrogen, carbon oxide, and methane were identified. The influence of pH and heat treatment on the physicochemical properties of titania-based materials during photoreduction of carbon dioxide have been studied. It was found that the photocatalysts prepared in neutral environment were shown to result in a higher content of hydrogen, carbon monoxide, and methane in the gas phase compared to photocatalysts obtained under basic conditions. The highest amounts of hydrogen were detected in the processes using photocatalysts heated in the microwave reactor, and double-heated photocatalysts.

## 1. Introduction

The use of fossil fuels, such as crude oil, natural gas, and hard coal, have increased the amount of pollutants emitted in the air. This condition has a direct impact on human health. Therefore, improving the quality of atmospheric air is one of the most serious challenges of modern civilization. The predominant gaseous air pollutants are nitrogen oxides (NO_x_), sulfur dioxide (SO_2_), volatile organic compounds (benzopyrenes), carbon monoxide (CO), and carbon dioxide (CO_2_). The increasing concentration of CO_2_ primarily causes the greenhouse effect to become stronger [1]. To reduce its emissions, CO_2_ capture from large point sources is used, which involves compressing and transporting CO_2_ to the storage site and storing it by permanently infusing it in rock formations (CCS, carbon capture and storage) [2]. An alternative to CCS is CCU (carbon capture and utilization) [3], which involves processing captured CO_2_, where carbon dioxide is used in various technological processes and industries.

One of the promising processes for CO_2_ utilization is photocatalysis, which can result in the production of various platform chemicals. Various types of compounds are used in the photocatalytic processes, including zinc or cadmium sulfide [4,5], cadmium selenide [6], vanadium (III) oxide [7], zinc oxide [8,9], or titanium (IV) oxide [10,11].

TiO_2_ is one of the most frequently used photocatalysts due to its high activity and physicochemical properties. It is cheap, non-toxic, chemically resistant, and relatively easy to process. Using this material as a photocatalyst, in both unmodified and modified forms, allows for reducing carbon dioxide to sustainable chemicals such as carbon monoxide (CO) [12,13], methanol (CH_3_OH) [14,15], methane (CH_4_) [16,17], ethylene (C_2_H_4_) [18,19], and formaldehyde (HCHO) [20,21], thereby also lowering the atmospheric CO_2_ concentration [22,23]. The selectivity and the amount of products obtained during the photocatalytic process depends on the properties, composition, and structure of the used photocatalyst. Since those parameters differ between titanium dioxide-based materials obtained via different preparation methods, overall photoactivity of a material depends on optimalization of the synthesis process.

Titanium dioxide-based photocatalysts can be prepared using different techniques, including sol–gel, hydrothermal or solvothermal, chemical vapor deposition, and physical vapor deposition [24,25]. One of the most popular methods is the sol–gel method, which includes the following steps: preparation of the initial homogeneous solution, hydrolysis, conversion of sol to gel, ageing, drying, and thermal treatment [11,26]. For the preparation of solutions, different precursors of TiO_2_, e.g., Ti(OC_2_H_5_), Ti(OC_3_H_7_)_4_, and Ti(OC_4_H_9_)_4_ [27], or TiOSO_4_ [28] and TiCl_4_ [29], are used, and different solvents, e.g., water, alcohol, and organic solvent, or a combination of two solvents with a certain ratio, are applied [30]. The addition of water initiates the hydrolysis process to a sol. Gelation involves condensation of hydroxyl and/or alkoxy groups leading to the release of water or alcohol and then polymerization [11]. To improve the strength of the links between particles, the ageing step, which takes a few hours or several days, is necessary before the drying step. To improve the structural stability, mechanical resistance, and to obtain the crystalline form of TiO_2_, a thermal treatment at different temperatures, usually in the range of 400 °C to 600 °C [11], is conducted.

Despite its advantages, the sol–gel method requires long post-heat treatments to remove the impurities, long processing time, and multiple steps [31], therefore modified versions of the sol–gel method have been developed. P. Reñones et al. [16], by combining the sol–gel method with electrospinning, obtained hierarchical assemblies of mesoporous TiO_2_ 1-D nanofibers with enhanced photocatalytic activity as compared to the particles obtained via the regular sol–gel method. Their hypothesis was that this change may be due to the presence of large interconnected interfaces in the obtained materials, contrasted to the random aggregation often found in TiO_2_ sol–gel samples.

To improve the physicochemical properties and photoactivity of photocatalysts, the synthesis can be conducted above room temperature and at a pressure higher than 1 atm, usually in autoclaves. When water is used as a solvent for the preparation of photocatalysts, these processes are defined as hydrothermal, while when organic solvents (e.g., n-butyl alcohol, ethanol, methanol, toluene) are applied, it is called the solvothermal method. G. Ren et al. [32] synthesized F-doped TiO_2_ using a hydrothermal technique. The results showed that these materials exhibit high activity for degrading Rose Bengal dye organic pollutants under visible light irradiation. B. Gomathi Thanga Keerthana et al. [33] successfully synthesized TiO_2_ with the hydrothermal method using NaOH and KOH as solvents. A. H. Mamaghani et al. [34] prepared TiO_2_ by hydro-/solvothermal synthesis, at relatively low temperature and above atmospheric pressure. These researches show that hydrothermal/solvothermal techniques have great potential in controlling and engineering properties of titanium dioxide-based photocatalysts.

Other enhancements to the sol–gel method include introducing the microwave-assisted solvothermal modification due to its superior mode of heating system [23,35]. This alternative is relatively novel method for producing photocatalysts, since microwave heating is an in situ energy conversion mode and radically differs from traditional heating processes [36,37]. M. Andrade-Guel et al. [38] synthesized titanium dioxide by microwave-assisted sol–gel and investigated the effect of the acid type (hydrochloric or acetic acid) used as catalyst on the phase transformation of TiO_2_. A single anatase phase was formed after a reaction time of only 15 min, shorter than that required for the conventional sol–gel method, when acetic acid was used as catalyst. When hydrochloric acid was used as catalyst, three titania polymorphs were detected: anatase, rutile, and brookite. C. M. Vladut et al. [39] studied the effect of thermal treatment on the structure and morphology of vanadium-doped ZnO nanopowders obtained using the same method. It was found that the temperature influenced the material morphology. The increase in the annealing temperature led to the grain growth. UV–Vis spectra showed that the absorption band intensity increased with the temperature of thermal treatment, and the sample that had been annealed at 650 °C had the highest absorption in the ultraviolet domain.

This technique, applied to the synthesis of titanium dioxide-based photocatalysts, allows for engineering certain properties of a materials. M. Akram et al. [35] studied the effect of the holding time of microwaves on the rate of the photodegradation reaction of methylene blue dye using TiO_2_ with a high surface area as a photocatalyst. They found that the increase in microwave holding time can enhance the degree of crystallinity and particle size of TiO_2_. A red shift in the band-gap values (E_g_) with an increase in the holding time of the microwave was also revealed. P. Ehi Imoisili et al. [37] synthesized vanadium and silver co-doped titanium oxide nanocatalysts through a microwave-assisted sol–gel route. The obtained materials demonstrated the reduced band gap, the large surface area, small particles with a narrow size distribution, and a significant photocatalytic ability in the degradation of methyl orange and methylene blue. G. Divya et al. [40] fabricated Zr-doped TiO_2_ mesoporous nanostructures using a microwave-assisted sol–gel approach, which demonstrated reduced crystallite size, band gap, and surface area and efficient photocatalytic activity on Bismark Brown red dye under visible light illumination. G. Divya et al. [40] also developed a facile and fast microwave irradiation method to synthesize uniform and well-defined nitrogen-doped TiO_2_ nanocatalysts through the microwave-assisted sol–gel method. The materials with the smallest crystallite size and a reduced band gap demonstrated the best photocatalytic ability under visible light, which enhanced the rate of degradation of indigo carmine dye. The same material also exhibits antibacterial and antifungal activity. L. Predoana et al. [41] used the microwave-assisted sol–gel method for synthesis of Zn- and Cu-doped TiO_2_ nanoparticles. The photocatalytic activity of the doped TiO_2_ nanopowders was tested for the degradation of methyl-orange dye. The results indicate that Cu doping using the described non-standard method increases the photoactivity of TiO_2_ in the visible-light range by narrowing the band-gap energy.

Materials prepared using a microwave-assisted process have also notably been used in the photocatalytic air purification research. A. May-Masnou et al. [42] fabricated small anatase titanium dioxide nanoparticles attached to larger anisotropic gold morphologies by a very fast and simple two-step microwave-assisted synthesis. The photocatalyst activity was evaluated in the photocatalytic production of hydrogen from water/ethanol mixtures in gas-phase at ambient temperature. M. Li et al. [43] used composites based on carbon quantum dots and TiO_2_ prepared using a facile microwave-assisted synthesis strategy for CO_2_ photoreduction. The amounts of CH_4_ and CO generated over composites were higher than those on pristine TiO_2_.

A lot of publications dealing with carbon dioxide capture and utilization have recently appeared, mainly dealing with photocatalysis at ambient conditions, but there are also some works about absorption of carbon dioxide in organic media, followed by transformation into useful products. Z.-Z. Yang et al. [44] developed an interesting and efficient binary system for carbon dioxide absorption and activation, composed of polyethylene glycol and an amidine or guanidine superbase. Activation of CO_2_ molecules led to the direct conversion of the captured CO_2_ into value-added chemicals or fuels. Another binary system was applied by A.-H. Liu et al. [45]; the system, composed of poly(ethylene glycol) in which N-substituted amino acid salts were dissolved, enabled CO_2_ to be reversibly absorbed at 1:1 stoichiometry. Carbamic acid was proposed as the absorbed form of CO_2_. The captured CO_2_ could be converted into oxazolidinones. J. Kothandaraman et al. [46] applied ruthenium and iron catalysts to convert carbon dioxide absorbed in amines in aqueous media into formate with a very good yield, up to 95% yield. Formate and methanol were products of carbon dioxide reduction reported by S. Kar et al. [47].

In this work, titanium dioxide was synthesized in a solvothermal reactor heated with microwaves under neutral or basic conditions. For comparison, the microwaved titanium dioxide materials were also prepared using a typical sol–gel technique under neutral or basic conditions. The photoactivity of these materials was studied through the photocatalytic reduction of carbon dioxide, resulting in carbon monoxide and methane as CO_2_ reduction products, and hydrogen formation from photocatalytic water splitting. The effect of changing pH during the preparation procedure and the mode of synthesis on the physicochemical properties and photocatalytic activity of titanium dioxide were compared and discussed.

## 2. Results and Discussion

### 2.1. Characteristics of the Obtained Materials 

**Figure 1:**
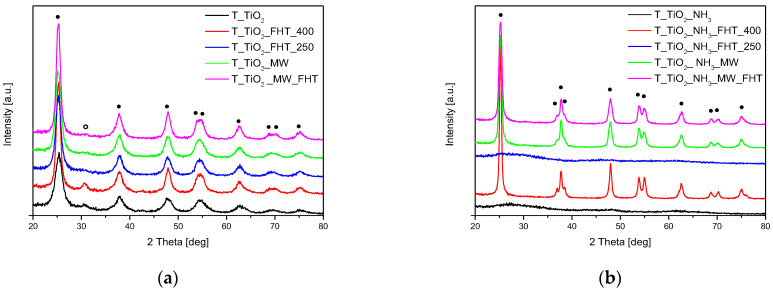
Diffraction patterns of the TiO_2_ materials obtained (**a**) without ammonia water and (**b**) with ammonia water. Reflections attributed to anatase are marked as •, and reflections attributed to brookite are marked as o.

X-ray diffraction (XRD) is a powerful technique used to analyze the crystal structure of materials, providing insights into their crystalline structure and phase composition. The XRD patterns exhibit diffraction peaks corresponding to specific crystallographic planes. For anatase, the peaks are typically observed around 25.3°, 37.8°, 48.0°, and 54.0° (2θ) [48]. In rutile, characteristic peaks are found at around 27.4°, 36.1°, 41.2°, and 54.3° (2θ) [48]. At lower preparation temperatures, typically below 400 °C, TiO_2_ is often found in the anatase phase, which has a tetragonal crystal structure. As the temperature increases, there might be a phase transition to the rutile phase, which has a more compact tetragonal structure. Analysis of the diffraction patterns of materials without ammonia water (Figure 1a) showed the same phase composition. In all samples, diffraction peaks were detected at about 25°, 38°, 48°, and 54°, which were assigned to the anatase structure (ICDD 01-073-1764) [49]. In addition to anatase, the peaks corresponding to brookite (ICDD 01-076-1937) [50] were identified in the samples. The presence of brookite is revealed by the peak at 30.8° [48,50]. At lower temperatures of TiO_2_ preparation, the XRD peaks may be broader, indicating smaller crystallite sizes. The results of the average crystallite size analysis are shown in Table 1. The anatase (101) peak was used to determine the crystallite size using Scherer’s formula. The crystallite size of TiO_2_ in the obtained samples increases with increasing preparation temperature. The titania sample obtained without heat treatment (T-TiO_2_) characterized the smallest crystallite size as being about 5 nm. When the temperature of preparation increased up to 250 °C, the crystallite size increased to 6.3 nm (T-TiO_2_-MW) and 6.8 nm (T-TiO_2_-FHT-250), no matter where the sample was heated, whether in the high-temperature furnace or microwave reactor. The largest crystallite sizes (8.8 nm and 9 nm) were observed for the samples heated at 400 °C in the high-temperature furnace (T-TiO_2_-FHT-400) and the samples heated in the microwave reactor and subsequently in a tubular furnace (T-TiO_2_-MW-FHT), respectively. The Williamson–Hall method [51,52] also confirmed the trend observed with Scherer’s formula, showing an increase in crystallite size with increasing preparation temperature (Table 1). The largest crystallite sizes observed indicated significant growth at higher temperatures. T-TiO_2_ had the smallest crystallite size at about 4.9 nm. The sample heated in the microwave reactor and furnace (T-TiO_2_-MW-FHT) had a crystallite size of about 9.6 nm. The lattice parameters a, b (≈3.79 Å), and c (≈9.49 Å) remained almost identical across all samples, with only slight variations observed between different heating methods and temperatures (Table 1). This suggests that the crystal structure of TiO_2_ is stable and not significantly altered by the preparation conditions. These findings indicate that the primary effect of increased preparation temperature is the growth of crystallite size, while the fundamental lattice structure of TiO_2_ remains largely unchanged.

The XRD spectra of the samples with ammonia water are shown in Figure 1b. In the case of samples not subjected to heating (T-TiO_2_-NH_3_) or heated at 250 °C in the tubular furnace (T-TiO_2_-NH_3_-FHT-250), the analysis of the diffraction patterns indicated that only an amorphous phase existed in the materials. Y.-H. Lin et al. observed that the anatase phase on pure and N-doped TiO_2_ samples started to appear after calcination at 200 °C and 300 °C, respectively [53]. The cited results and the results obtained in this work imply that the addition of ammonia water during the preparation of TiO_2_ releases the phase transformation. In other samples, similar to materials prepared without ammonia water, the presence of the anatase (ICDD 01-073-1764) phase was confirmed, whereas no brookite phase was recorded in the studied materials. It is worth noting that, when comparing the two samples obtained at the same temperature (250 °C), a crystalline phase was not observed for the material prepared at high-temperature furnace (T-TiO_2_-NH_3_-FHT-250), the sample synthesized at microwave rector (T-TiO_2_-NH_3_-MW). It was also found that the addition of ammonia water led to an increase of average crystallite size in the obtained samples (Table 1), and the calculated values were higher than those for the samples prepared without ammonia water. However, the average crystallite size of TiO_2_ increases with increasing temperature treatment as well (Table 1). For the material heated at 400 °C in the tubular furnace (T-TiO_2_-NH_3_-FHT-400), the average crystallite size equaled 21.9 nm, while the materials synthesized in the but was for microwave reactor (T-TiO_2_-NH_3_-MW) possessed smaller crystallites and their size was about 14.3 nm. For the sample that was first treated in the microwave reactor and then reheated in the furnace (T-TiO_2_-NH_3_-MW-FHT), the average crystallite size equaled 13.5 nm. This value was more similar to that obtained in the reactor than in the furnace, what indicates that the crystallites were formed in the microwave reactor, and that the reheating did not change crystallite size any further.

The results obtained by the Williamson–Hall method show that the crystallite size in all samples with the addition of ammonia water are the same, approximately 16 nm (Table 1). The strain in the materials also varies slightly with the preparation method. The observed strain in samples T-TiO_2_-NH_3_-FHT-400 and T-TiO_2_-NH_3_-MW is 0.0021 and 0.0022, except for the sample treated both in the microwave reactor and furnace, which exhibited a slightly lower strain of 0.0015 (Table 1). The lattice parameters a, b = 3.78 Å, and c = 9.49 Å are consistent across all samples, indicating minimal lattice distortion regardless of the preparation method (Table 1). The consistent lattice parameters across samples indicate stable crystal structure formation under the different treatment conditions.

Textural properties of the synthesized materials were also studied. Based on the adsorption/desorption isotherms, the specific surface area (S_BET_) and pore volumes were determined, and the calculated values are shown in Table 2.

**Figure 2:**
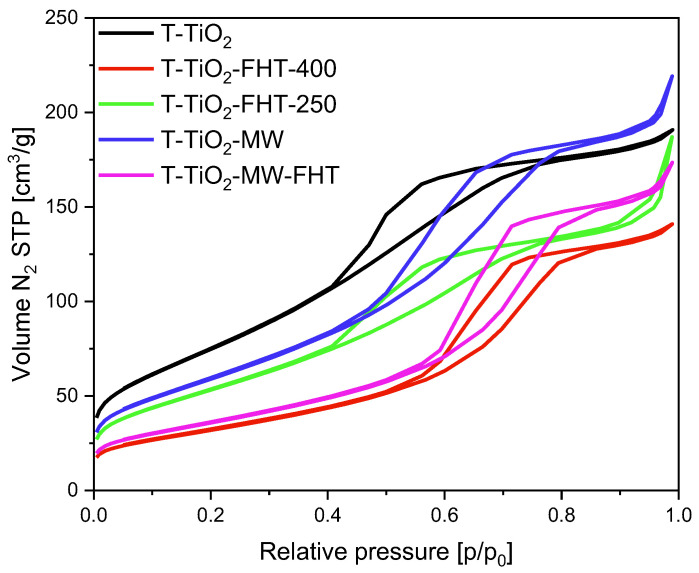
N_2_ adsorption/desorption isotherms of the materials prepared without ammonia water.

The adsorption/desorption isotherms obtained for the samples without the addition of ammonia water are presented in Figure 2. According to IUPAC classification, they are a combination of type II and IV isotherms, indicating the presence of macro- and mesopores in the materials. In addition, the presence of a H2(b) hysteresis loop is characteristic for mesoporous materials and indicates a high distribution of pore neck size. For the materials obtained at lower temperatures, namely T-TiO_2_, T-TiO_2_-MW, and T-TiO_2_-FHT-250, a hysteresis loop occurs from 0.4 to 0.75 relative pressure. The increase of the preparation temperature resulted in the shift of the hysteresis loop towards higher values of relative pressure, from 0.6 to 0.85 for the materials obtained at 400 °C (T-TiO_2_-FHT-400 and T-TiO_2_-MW-FHT), pointing to the presence of larger pores in these samples.

The surface area of the samples prepared without adding ammonia water ranged from 281 m^2^/g for T-TiO_2_ to 117 m^2^/g for T-TiO_2_-FHT-400. The higher the treatment temperature, generally the lower the final surface area of the TiO_2_. The same tendency can be noticed for the values of micropores. The samples prepared at the temperature ≤250 °C express volume of micropores from 0.04 cm^3^/g for the sample T-TiO_2_ to 0.023 cm^3^/g for T-TiO_2_-MW. In contrast, the volume of micropores for all samples prepared at 400 °C was around 0.01 cm^3^/g.

**Figure 3:**
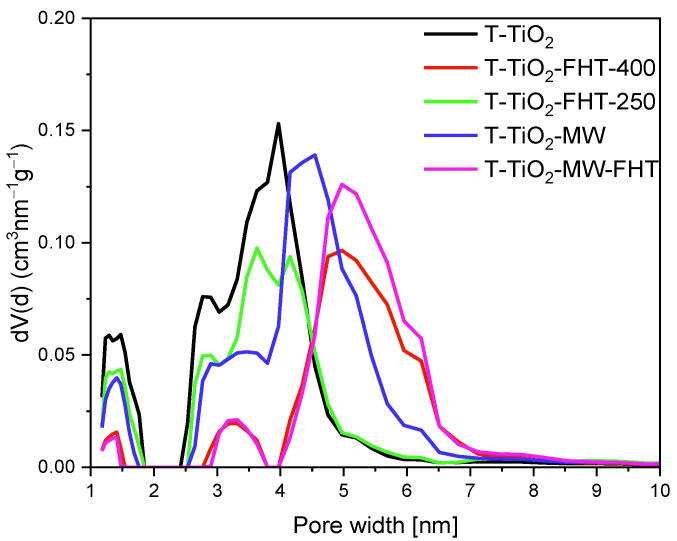
Pore size distributions of the materials prepared without ammonia water derived from the DFT calculation method.

For further investigation of the porosity of characterized materials, the DFT method derived from N_2_ adsorption/desorption isotherms at −196 °C was used, and the results are presented in Figure 3. For all materials, two ranges of pore sizes can be distinguished: 1 to 2 nm and 2.5 to 7 nm. The treatment of TiO_2_ at lower temperatures resulted in a higher contribution of pores in the range from 1 to 2 nm and from 2 to 5 nm. Application of higher temperatures during preparation resulted in the diminishment of the content of pores from 1 to 2 nm. Moreover, a considerable shift towards higher content of pores that are larger in size has been noticed. The mean pore width of the mesopores for the samples prepared at lower temperatures distributes from 4 nm for the sample T-TiO_2_ to 4.5 nm for the sample T-TiO_2_-MW, whereas samples prepared at higher temperatures show larger pores (around 5 nm for T-TiO_2_-FHT-400 and T-TiO_2_-MW-FHT samples).

**Figure 4:**
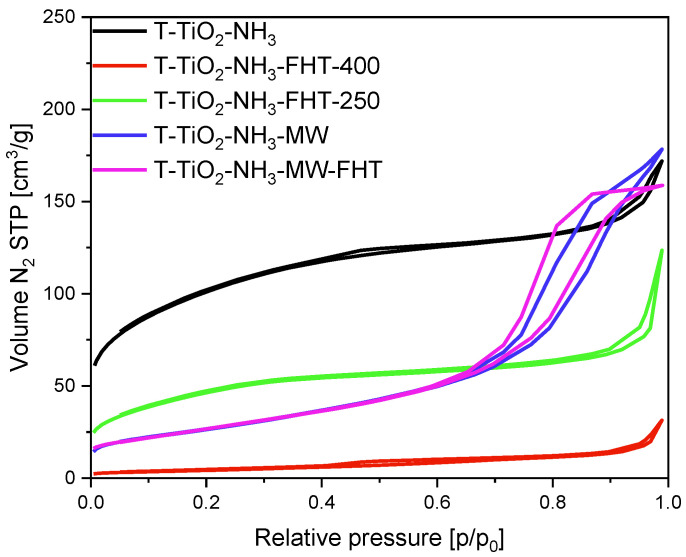
N_2_ sorption isotherms of the materials prepared with ammonia water.

Another set of samples was prepared using ammonia water, and the N_2_ sorption isotherms for these samples are presented in Figure 4. The isotherms of samples T-TiO_2_-NH_3_, T-TiO_2_-NH_3_-FHT-250, and T-TiO_2_-NH_3_-FHT-400 are a mixture of type I and II, indicating the presence of micro- and mesopores. On the other hand, the isotherms recorded for T-TiO_2_-NH_3_-MW and T-TiO_2_-NH_3_-MW-FHT are of type II characteristic for mesoporous materials, with H3 hysteresis loop associated with the presence of macropores.

Although the tendency to diminish the surface area values with increased treatment temperature is the same as for samples without adding ammonia water, samples with NH_3_·H_2_O generally express lower S_BET_ values. The specific surface area of the not thermally treated sample T-TiO_2_-NH_3_ was the highest and reached 342 m^2^/g. Treatment of TiO_2_ in the tubular furnace at 250 °C caused a reduction of S_BET_ to 163 m^2^/g for T-TiO_2_-NH_3_-FHT-250. When the microwave reactor was applied, and the temperature of the process was also around 250 °C, the surface area of sample T-TiO_2_-NH_3_-MW was 98 m^2^/g. At the highest temperature, 400 °C, the surface area of the obtained TiO_2_ was reduced to 16 m^2^/g for T-TiO_2_-NH_3_-FHT-400. Together with the surface area, a considerable loss in micropore values was noticed, from 0.11 cm^3^/g for T-TiO_2_-NH_3_ to 0.002 cm^3^/g for T-TiO_2_-NH_3_-FHT-400 0.05 cm^3^/g.

**Figure 5:**
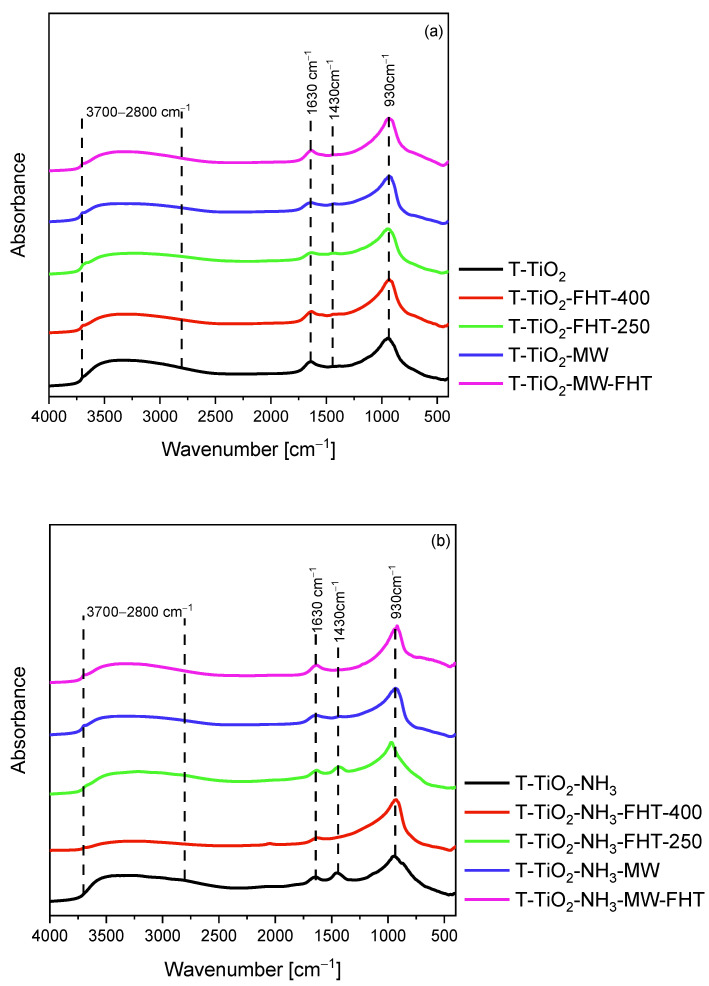
DRIFT spectra of TiO_2_ prepared without (**a**) or with ammonia water (**b**).

The DRIFT spectra presented in Figure 5 show the surface characterization of the photocatalysts. All the spectra present some characteristic peaks for pure TiO_2_ samples. A wide band from 3700 cm^−1^ to 2800 cm^−1^ corresponds to the stretching mode of surface −OH groups, while a narrow band located at 1620 cm^−1^ is characteristic of the molecular water bending mode [54]. All materials also exhibit the intensive band at around 930 cm^−1^, which corresponds to the self-absorption of titania [55]. The samples also have characteristic bonds related to the vibrational modes of organic species, such as the methyl group located at 1430 cm^−1^. This band is observed due to residual ethanol species during the sol–gel process [56]. The intensity of these bands is considerably enhanced for some TiO_2_ samples obtained with ammonia water (T-TiO_2_-NH_3_, T-TiO_2_-NH_3_-FHT-250, and T-TiO_2_-NH_3_-MW) because it overlaps with the characteristic peak of NH_4_^+^ located at around 1460 cm^−1^ [57,58]. This phenomenon is not observed in the case of samples obtained with ammonia water and annealed at 400 °C, indicating the desorption of ammonium species during heat treatment at an elevated temperature. B. Tryba et al. [59] and L. Zhu et al. [60] noted a similar situation. L. Zhu et al. [60] explained this with asymmetric and symmetric deformation vibration of NH_4_^+^, which are thermally unstable.

**Figure 6:**
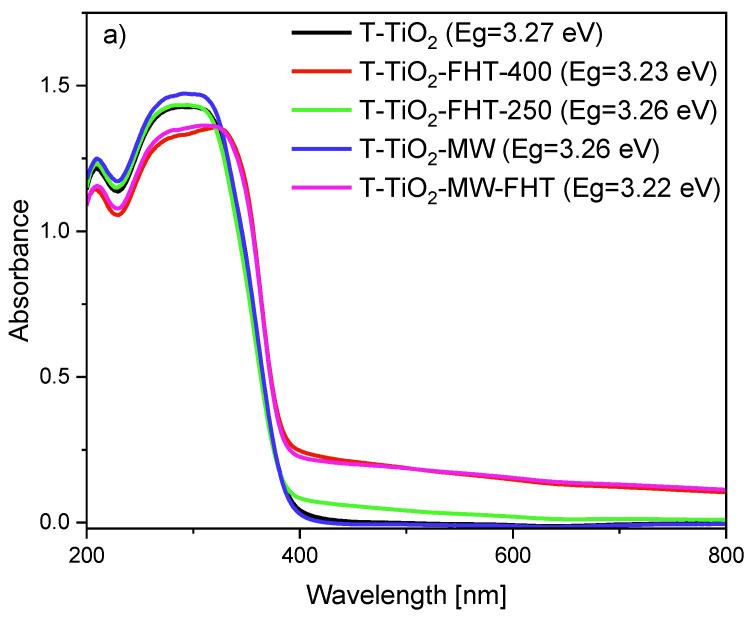

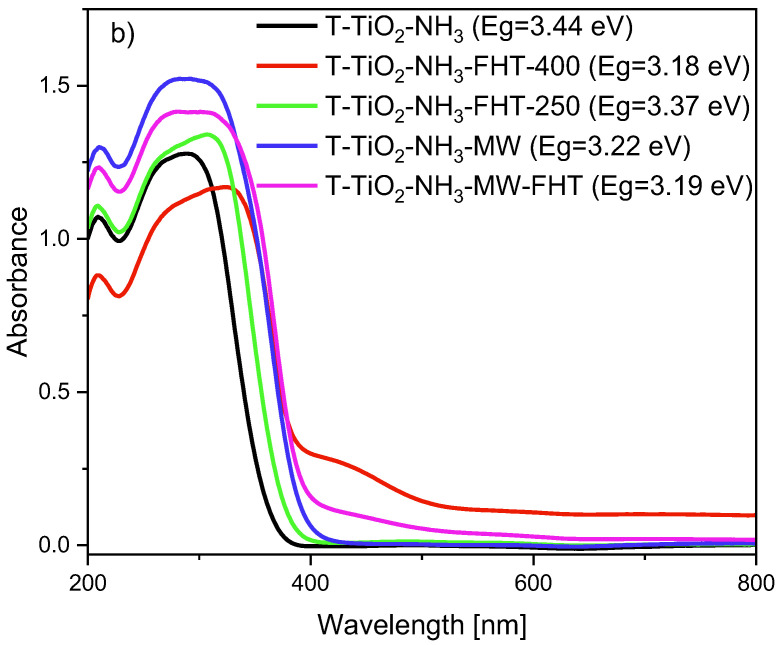
UV–Vis/DR spectra of TiO_2_ prepared without (**a**) or with ammonia water (**b**).

The optical absorbance spectra of TiO_2_ obtained with ammonia water or without ammonia water are presented in Figure 6. All of the tested samples show a strong absorption at wavelengths shorter than 400 nm, typical for the intrinsic interband absorption of titania. Additionally, a broad absorption band in the range from 400 nm to 800 nm was observed for both obtained samples without ammonia water (T-TiO_2_-MW-FHT and T-TiO_2_-FHT-400), as well as with ammonia water (T-TiO_2_-NH_3_-MW-FHT) and T-TiO_2_-NH_3_-F-400). Further calcination of the samples at 400 °C caused the color change of photocatalysts. In case of samples obtained without ammonia water, the color changed from white to greyish due to the presence of residual ethanol species used for preparation, while for materials prepared with ammonia water, the color changed to yellowish due to the presence of nitrogen. H. Suda et al. [61] concluded that this phenomenon is related to the concentration rate of nitrogen atoms in TiO_2_-_x_N_x_ particles. Furthermore, a redshift of absorption edges into the visible region is observed for T-TiO_2_-NH_3_-MW-FHT and T-TiO_2_-NH_3_-FHT-400 samples. The E_g_ values for these materials were 3.18 and 3.19 eV, respectively, compared to pure TiO_2_ (3.27 eV). The noticeable shift to the visible light region can be ascribed to either the nitrogen doping and/or sensitization by a surface-anchored group [62,63]. Considering that nitrogen groups for these photocatalysts are not observed on DRIFT spectra, the redshift can be attributed to the substitutional N-doping, for example, for not visible on FT-IR/DRS more thermally stable coordinated NH_3_ molecules. Based on this phenomenon, the tested photocatalyst could be active under visible light irradiation.

All the prepared samples were tested in the photocatalytic carbon dioxide reduction process. Hydrogen, methane, and carbon monoxide were detected in the gas phase. Although hydrogen is not a direct product of CO_2_ photoreduction, but a result of the water splitting process, it is a valuable product and an essential component for CO_2_ conversion.

### 2.2. CO_2_ Photoreduction Processes Involving Titanium Dioxide Samples Obtained under Neutral and Basic Conditions

The samples of titanium dioxide without the addition of ammonia water during the preparation process were tested in the photocatalytic carbon dioxide reduction process, and the amounts of hydrogen, methane, and carbon monoxide in the gas phase were analyzed.

**Figure 7:**
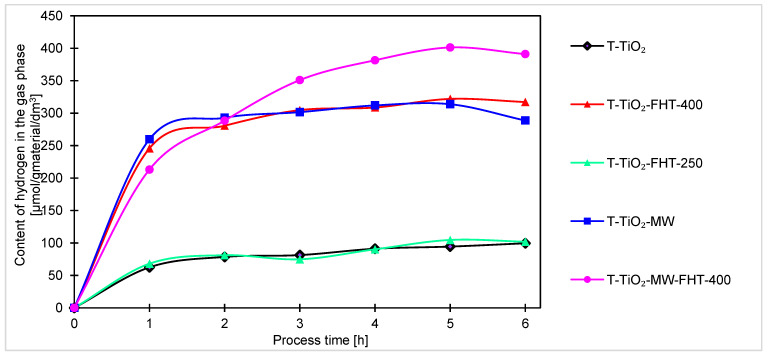
Comparison of hydrogen content obtained by photocatalysis of samples prepared without ammonia water.

The hydrogen contents in the gas phase during the 6 h of the process are presented in Figure 7.

Comparing the graphs of hydrogen content in the gas phase of the tested system, the samples can be divided into two groups differing in photocatalytic activity. The first group includes samples prepared in the microwave reactor (T-TiO_2_-MW, T-TiO_2_-MW-FHT) and the sample heated in the tubular furnace at 400 °C (T-TiO_2_-FHT-400). The second group includes those that did not undergo any of these modifications.

Among the samples prepared without the addition of ammonia water, the highest hydrogen content in the gas phase was also obtained using the doubly heated (in the reactor and then in the tubular furnace) T-TiO_2_-MW-FHT sample, and it was 391 μmol/g_material_/dm^3^. The sample obtained by heating only with the microwave reactor and the sample obtained by annealing only in an oven at 400 °C had a similar initial increase in hydrogen content, but reached only 288 μmol/g_material_/dm^3^ and 317 μmol/g_material_/dm^3^, respectively. They reached a plateau after just over an hour of running the process. The plateau for these samples occurred at a value representing about 80% of the maximum hydrogen content reached with the doubly annealed sample.

The position of samples T-TiO_2_-MW (288 μmol/g_material_/dm^3^) and T-TiO_2_-FHT-250 (102 μmol/g_material_/dm^3^) allows us to conclude that the differences in photocatalytic activity of the catalysts modified in the microwave reactor again are not due to temperature differences, but due to the influence of the microwaves used in the reactor. For the unheated sample (T-TiO_2_), only 99 μmol/g_material_/dm^3^ of hydrogen was obtained.

**Figure 8:**
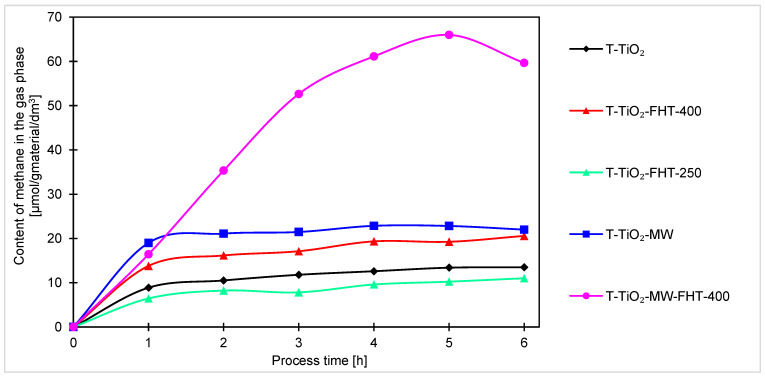
Comparison of methane content obtained by photocatalysis of samples prepared without ammonia water.

The graph concerning methane production is shown in Figure 8. Methane was obtained in the smallest concentration out of all conversion products.

The highest amount of methane was also obtained for the sample annealed twice (in the microwave reactor and then in the tubular furnace), reaching 59 μmol/g_material_/dm^3^. The amount of methane obtained with this sample is nearly three times higher than that of T-TiO_2_-MW (22 μmol/g_material_/dm^3^). The remaining samples showed comparable photocatalytic activities, yielding between 11 μmol/g_material_/dm^3^ and 20 μmol/g_material_/dm^3^ of methane after 6 h.

**Figure 9:**
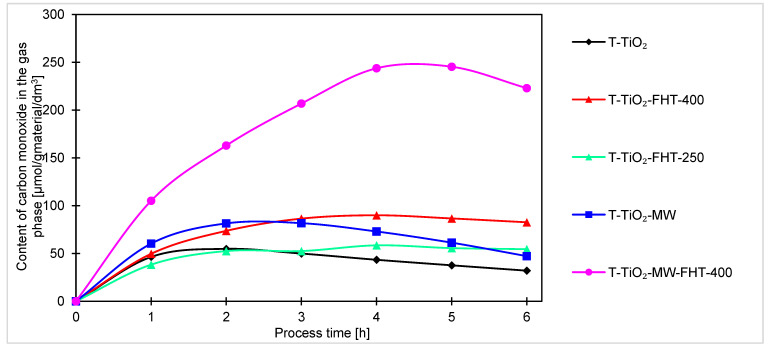
Comparison of carbon monoxide content obtained by photocatalysis of samples prepared without ammonia water.

A graph of carbon monoxide production is shown in Figure 9. As can be seen, the trends in the product yield curves are similar to those for methane production.

The highest amount of CO was obtained in the process using a doubly annealed sample (T-TiO_2_-MW-FHT), and it reached 223 μmol/g_material_/dm^3^. For the other samples, the CO content in the gas phase after 6 h of the process ranged from 32 μmol/g_material_/dm^3^ to 82 μmol/g_material_/dm^3^.

**Figure 10:**
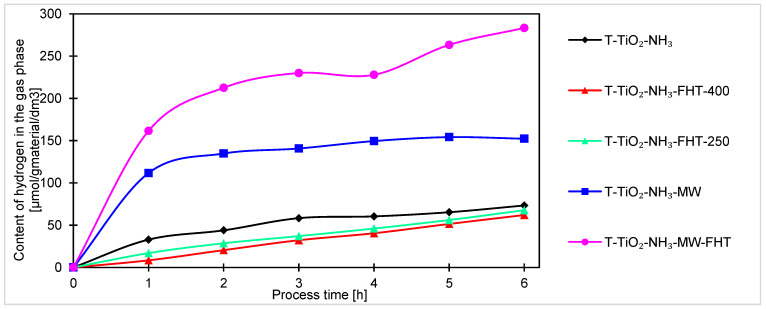
Comparison of hydrogen content obtained during photocatalytic carbon dioxide reduction using titanium dioxide prepared with the addition of ammonia water.

The hydrogen contents in the gas phase during the 6 h of the process, using titanium dioxide with the addition of ammonia water, are presented in Figure 10. Comparing them, the samples can be divided into two groups differing in photocatalytic activity. The first group, with much higher hydrogen production rate, includes samples modified using the microwave reactor (T-TiO_2_-NH_3_-MW and T-TiO_2_-NH_3_-MW-FHT), which is similar to the samples without addition of ammonia water. The second group comprises those that have not undergone such modification and were heated in the high-temperature furnace (T-TiO_2_-NH_3_-FHT-250, T-TiO_2_-NH_3_-FHT-400) or were not heated at all (T-TiO_2_-NH_3_).

Among the samples prepared with the addition of ammonia water, the highest hydrogen content in the gas phase was achieved for the sample heated first in the microwave reactor and then in the tubular furnace, T-TiO_2_-NH_3_-MW-FHT, and it was 283 μmol/g_material_/dm^3^. This sample was calculated to have the smallest average crystallite size among the whole group of samples prepared with the addition of ammonia water, which may be one of the reasons why the observed photocatalytic activity was the highest. This suggestion is supported by the fact that the sample obtained by heating only in the microwave reactor (having both the second lowest size of crystallites among the “NH_3_-samples” and very comparable BET surface value to the T-TiO_2_-NH_3_-MW-FHT) also had similarly higher photoactivity relative to the samples prepared without the use of microwave reactor [64]. After just the first hour of running the process, the amount of hydrogen obtained in the process catalyzed by T-TiO_2_-NH_3_-MW was more than two times higher than the amount of hydrogen obtained in the process catalyzed by T-TiO_2_-NH_3_-FHT-400 (the catalyst heated using only using the tubular furnace).

Comparison of T-TiO_2_-NH_3_-MW with the T-TiO_2_-NH_3_-FHT-250 sample (heated in the high-temperature furnace but at a temperature close to the inside of the microwave reactor) allows us also to conclude that the differences in photocatalytic activity of the two catalysts are not due to temperature differences, but to the influence of the microwaves used in the reactor [65], which helped to nucleate small crystallites with relatively high surface area. The hydrogen amounts in the gas phase after 6 h of the process for those samples were 152 μmol/g_material_/dm^3^ and 67 μmol/g_material_/dm^3^, respectively.

The three samples, which were not prepared in the microwave reactor, showed photocatalytic activity toward hydrogen at a similar level without characteristic differences. In this case, the range of hydrogen amounts was 62–73 μmol/g_material_/dm^3^. Taking X-ray diffraction results into account, it can be stated that the crystallization degree of the samples did not significantly impact their photoactivity because only the sample T-TiO_2_-NH_3_-FHT-400 was crystallite, but the remaining two titania samples, denoted T-TiO_2_-NH_3_ and T-TiO_2_-NH_3_-FHT-250, consist of an amorphous phase.

**Figure 11:**
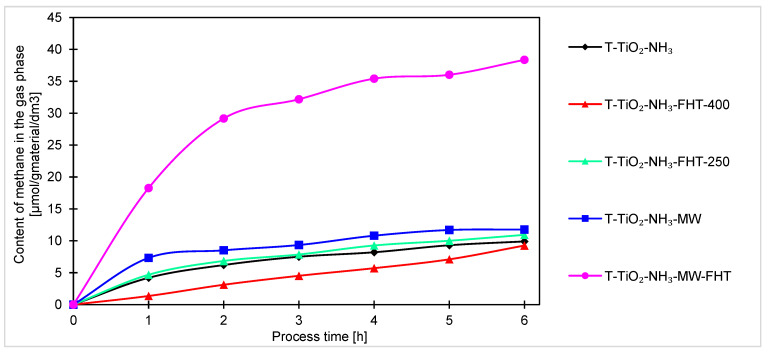
Comparison of methane content obtained by photocatalysis of samples prepared with ammonia water.

Another identified product of the process was methane. The graph concerning this compound is shown in Figure 11. As with the samples prepared without ammonia water, methane was produced at the lowest yield out of the three products.

The effect of microwaves on increasing the photocatalytic activity of the material can also be seen by measuring the amount of methane in the process. The highest amount of methane was obtained for the sample heated twice (T-TiO_2_-NH_3_-MW-FHT). After 6 h of the process, it was 38 μmol/g_material_/dm^3^, which was much more as for the second-best sample T-TiO_2_-NH_3_-MW, for which 12 μmol/g_material_/dm^3^ of methane was obtained.

While measuring the photoactivity of titania in the process of CO_2_ reduction towards methane, the samples heated only in the tubular furnace obtained a similar amount of methane, in the range of 9.3–10.9 μmol/g_material_/dm^3^. At the same time, it can be seen that the sample T-TiO_2_-NH_3_ (not heated) exhibited similar activity and yielded 9.9 μmol/g_material_/dm^3^ of methane during the process.

**Figure 12:**
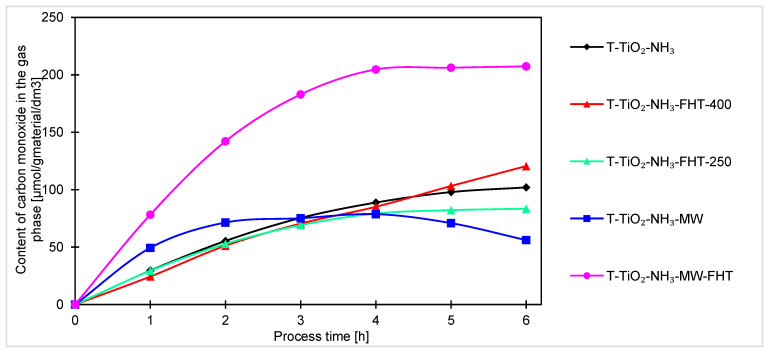
Comparison of carbon monoxide content obtained by photocatalysis of samples prepared with ammonia water.

CO is basically the first product of CO_2_ photoreduction. It can also further convert toward CH_4_. However, due to the much lower energy requirements for obtaining CO compared to methane, its amount is much higher in the gas phase after 6 h of irradiation. The graphs concerning carbon monoxide content in the gas phase in the processes using “NH_3_-samples” are presented in Figure 12.

The strong impact of treatment procedure on enhancing the photocatalytic properties of titanium dioxide can be seen in the process of photoreduction of carbon dioxide to carbon monoxide. Analysis of Figure 12 shows that samples annealed in a microwave reactor and next in the high-temperature furnace at 400 °C (T-TiO_2_-NH_3_-MW-FHT) obtained more than twice the amount of carbon monoxide in the gas phase as compared to the others, and it was 207 μmol/g_material_/dm^3^. For the other materials, the amounts were between 56 μmol/g_material_/dm^3^ and 120 μmol/g_material_/dm^3^.

It should be noted that at a certain point in the process, CO production stabilizes and sometimes even decreases. This may be related to either faster kinetics of a subsequent reaction between CO and H_2_, or the occupation of active sites by products or unreacted CO_2_, which prevents the reaction from proceeding further [66]. Only in the case of the sample annealed in the tube furnace (T-TiO_2_-NH_3_-FHT-400) did the CO content continuously increase, suggesting that saturation of the active sites in the T-TiO_2_-NH_3_-FHT-400 photocatalyst occurs slower than for the other samples.

Generally, for all samples (obtained under neutral and basic conditions), the highest increase of products occurs after the first hour of the process. Considering the course of the photocatalytic reaction, produced samples can be divided into two sets. In one way, after the first hour of the process, the reaction reaches equilibrium, and no or very small increment of products can be noticed. Another set of samples is characterized by a steady increase of products over time, indicating that these samples are less active and require more time to stabilize. As the processing time increases, the increment of products decreases, suggesting the formation of products that were unfortunately not investigated in this research. The decreases, and thus the change in the kinetics of carbon monoxide, methane, and hydrogen generation observed in the final hours of the process, can be generally explained by the poisoning mechanism of the photocatalysts’ surface by intermediates and byproducts of the reaction, as examined and described by H. Li et al. [67] and P. Haghighi et al. [68]. In our case (as shown in Figure 7, Figure 8 and Figure 9), the formation of CO, CH_4_, and H_2_ (as a side reaction of photocatalytic water splitting) occurs with different intensities, depending on the type and composition of the photocatalyst. This generally results from the reaction mechanism: CO (formed during the CO_2_ photoreduction) accumulates on the photocatalyst surface through chemical binding to the active sites. Next, the reduction intermediates may be various forms of carbon deposits, forming agglomerates and blocking the active surface of the photocatalyst, thus suppressing the efficiency of CO_2_ photoreduction and H_2_ evolution. As a result, the hydrogenation of carbon deposits to methane slows down, and the primary reaction of photocatalytic water splitting is inhibited. Moreover, in a closed circulation system, the amount of oxygen from water splitting increases to 1%, which may result in the undesirable oxidation of hydrogen, methane, and carbon monoxide.

### 2.3. Selectivity of Titanium Dioxide Samples Obtained under Neutral and Basic Conditions in the Carbon Dioxide Photoreduction Process

During the overall photocatalytic process, reactions (1) and (2) are often attributed to the formation of carbon monoxide and methane in the gas phase [17]:

CO_2_ + 2H^+^ + 2e^−^ → CO + H_2_O (1)

CO_2_ + 8H^+^ + 8e^−^ → CH_4_ + 2H_2_O (2)

The hydrogen evolution is associated with a side reaction of photocatalytic water splitting as presented in reaction (3) [69]:

2H^+^ + 2e^−^ → H_2_ (3)

Using these reactions as possible parallel pathways for CO_2_ conversion, the selectivity of process can be calculated for each of the described photocatalysts (Table 3).

Samples obtained in the basic conditions all represent significantly higher selectivity values towards carbon monoxide than those prepared using neutral conditions when comparing selectivity values of carbon dioxide conversion. This difference may be attributed to the difference in the probability of surface chemisorption shown by both sample groups. The addition of ammonia water during the preparation step reduced the BET surface area of samples as compared to their counterparts prepared in the neutral conditions, which generally lowers carbon dioxide binding capabilities of the samples of those samples, thereby favoring the occurrence of less complex (requiring less electrons) reaction pathway. This hypothesis is further confirmed by the overall worse photocatalytic activity of the samples prepared using ammonia water.

In both sets of samples, it can be observed that samples that have undergone the microwave treatment (MW) are characterized by a higher selectivity of conversion towards methane than their counterparts heated only using the high-temperature furnace (FHT). Coupling this with the previous observation that microwave-assisted treatment allows for obtaining more porous samples (as compared to the samples annealed in the same temperature, but in tube furnace) and the fact that kinetic diameter of methane molecule (380 pm) is over 15% bigger than kinetic diameter of carbon dioxide molecule (330 pm) [70,71,72], it can be suggested that higher porosity of the sample positively affects its selectivity of CO_2_ conversion towards methane. This claim can be confirmed by the fact that the highest selectivity towards methane was exhibited by the samples T-TiO_2_-MW and T-TiO_2_-NH_3_-MW (31.8% and 17.4%, respectively), which had the highest total porosity value (TPV) among their respected groups.

Overall profiles of the selectivity towards measured products are similar for all the samples; in all cases, H_2_ has the highest calculated selectivity value, most commonly followed by CH_4_ and then by CO. Exceptions to this trend are presented by the samples annealed in the furnace; however, these changes are small and do not affect the trend that all of the samples are most conducive for photocatalytic water decomposition processes as compared to alternative reactions (1) and (2). Out of all of them, T-TiO_2_-MW is the best tailored to obtain mainly H_2_ during the process (selectivity of 68.1% towards H_2_).

Incidentally, the sample that obtained the highest amount of hydrogen during the process (T-TiO_2_-MW-FHT) had the lowest relative selectivity among the compared group of samples. This sample was also simultaneously characterized by the highest value of calculated strain and highest average crystallite size diameter (based on Table 1). Coupling that fact with the decrease in a parameter and increase in c parameter of a unit cell (as compared to the other samples in this group), it may be concluded that achieving a more elongated shape of an unit cell is beneficial for both the overall photocatalytic process and the enhanced selectivity towards all measured products, due to the presence of a shape more prone to creating active sites. It means therefore that doubly treated material is the best multipurpose photocatalyst in this group of samples.

Contrary to the selectivity profiles represented by the samples prepared in the neutral conditions, the distribution of selectivity for “NH_3_-type samples” show two distinct types of profiles for the selectivity towards measured products. The first type of selectivity distribution profile (with the highest selectivity toward CO) can be found in the samples treated only in the high-temperature furnace and the non-treated one, while the second type (with the highest selectivity towards H_2_) is characteristic of the samples that have undergone treatment in the microwave-assisted reactor.

The first type of selectivity profile is characteristic for samples that obtain larger quantities of carbon monoxide in the gas phase during the process, while simultaneously reducing the amount of both hydrogen and methane being produced. Therefore, this type of profile suggests that adding ammonia water in conjunction with the annealing treatment leads to obtaining photocatalyst tailored slightly better toward obtaining carbon monoxide via the CO_2_ conversion and not hydrogen via water-splitting.

However, the sample that obtained the highest amount of CO (as well as both H_2_ and CH_4_) during the process was T-TiO_2_-NH_3_-MW-FHT, representing the second type of the selectivity profile, which again proves that the material, which underwent both microwave high-pressure treatment as well as annealing (double treatment), is the best photocatalyst overall in this group of samples.

## 3. Materials and Methods

### 3.1. Materials Preparation

Two series of titanium dioxide samples were obtained via hydrolysis of titanium (IV) isopropoxide using a sol–gel method.

In the case of the first series, 50 mL of distilled water was added dropwise to the glass beaker containing 20 mL of titanium (IV) isopropoxide (TTIP) and 5 mL of 99.9% ethanol. To change pH, ammonia water (25% NH_3_∙H_2_O) was added until the pH value was equal to 10. Regardless of whether ammonia water was added or not, the whole was continuously stirred for 24 hand next was left to age for another 24 h. Finally, the material was dried in a forced-air dryer at 80 °C and ground in an agate mortar to a uniform consistency. The obtained samples, without or with ammonia water, were denoted as T-TiO_2_ and T-TiO_2_-NH_3_, respectively. Optionally, after the ageing process, T-TiO_2_ or T-TiO_2_-NH_3_ samples were placed into a Teflon vessel and transferred to a microwave reactor (ERTEC MAGNUM II, Wrocław, Poland). The process was conducted for 15 min under a pressure of 40 bar. Finally, the material was dried in a forced-air dryer at 80 °C and grounded in an agate mortar to a uniform consistency. The obtained sample was denoted as T-TiO_2_-MW or T-TiO_2_-NH_3_-MW.

T-TiO_2_ or T-TiO_2_-NH_3_ samples were also subjected to a heat treatment. For this purpose, the obtained powder was transferred into a quartz boat and placed in a high-temperature furnace with argon flow. Air was replaced through a 30 min argon flush (10 L/h) before the process was started: the annealing of the sample lasted for 60 min at 250 °C or 400 °C, with an initial heating rate of 10 °C/min. Afterwards, the samples were cooled to room temperature in argon atmosphere. The obtained materials were washed in a vacuum filtration set, which was performed until the pH achieved a value of about 7. Then, the sample was re-dried, ground in an agate mortar to a uniform consistency, and transferred into a suitable container. Depending on the heating temperature, 250 °C or 400 °C, the samples were denoted as T-TiO_2_-FHT-250 and T-TiO_2_-NH_3_-FHT-250 or T-TiO_2_-FHT-400 and T-TiO_2_-NH_3_-FHT-400, respectively.

In addition to the treatment in the high-temperature furnace, the samples obtained using a microwave reactor, denoted as T-TiO_2_-MW or T-TiO_2_-NH_3_-MW, were also subjected to the procedure described above. Samples that were heated at 400 °C were denoted as T-TiO_2_-MW-FHT or T-TiO_2_-NH_3_-MW-FHT.

In Table 4 summarizes all samples and basic experimental conditions.

### 3.2. Material Characteristics

The phase composition of the prepared samples was determined with X-ray diffraction using Cu Kα radiation (λCu Kα = 0.1540 nm) on an Empyrean (Panalytical, Malvern, UK). The identification of the crystalline phases was performed using HighScore+ and the ICDD PDF-4+ 2015 database. The crystallite size was calculated according to the Scherrer equation based on the X-ray powder diffraction patterns:

$$D=\frac{k\lambda }{\beta cos\theta }$$

where *D* is the average crystallite size in the direction perpendicular to the (*hkl*) reflection plane; *k* is a constant close to unity, dependent on the shape of the crystallite; *λ* is the X-ray wavelength; *β* is the peak broadening; and *θ* is the XRD peak position.

The analysis of the XRD diffractograms by the Williamson–Hall method [52] have been carried out:

$$\beta_{hkl}cos\theta =\frac{k\lambda }{D}+4\Sigma sin\theta$$

where *D* is grain size as determined from the Williamson–Hall plot; *k* is a constant close to unity, dependent on the shape of the crystallite; *λ* is the X-ray wavelength; *β* is the peak broadening; and *θ* is the XRD peak position.

The lattice parameters (*a* and *c*) were calculated from the XRD data using Bragg’s law applied for the tetragonal crystal symmetry [73]:

$$\frac{1}{d^{2}}=\frac{h^{2}+k^{2}}{a^{2}}+\frac{l^{2}}{c^{2}}$$

where *h*, *k*, and *l* are Miller indices and *d* is the inter planar spacing of the crystal lattice.

To perform the low-temperature nitrogen adsorption/desorption studies, a QUADRASORB evo^TM^ gas sorption automatic system (Quantachrome Instruments, Anton Paar, Graz, Austria) was used, together with a MasterPrep multi-zone flow/vacuum outgassing system under a vacuum of 1 × 10^−5^ mbar from Quantachrome Instruments (Boynton Beach, FL, USA). On the basis of the obtained adsorption/desorption isotherms, the specific surface area (S_BET_) and pore volumes of the materials were determined. Prior to analysis, 150 mg of the material was weighed and pre-dried at 90 °C in a laboratory dryer. The dried samples were transferred into measuring cells and degassed using MasterPrep (Quantachrome Instruments, Boynton Beach, FL, USA) at 100 °C for 12 h. The Brunauer–Emmett–Teller (BET) equation was used to determine the surface area (S_BET_) in the relative pressure range of 0.05–0.2. The total pore volume (V_total_) was calculated from the volume of nitrogen held at the highest relative pressure (p/p_0_ = 0.99). The volume of micropores (V_micro_ < 2 nm) with dimensions smaller than 2 nm was calculated as a result of integrating the pore volume distribution function using the DFT method; mesopore volume (V_meso_) with dimensions from 2 to 50 nm was calculated from the difference of the total pore volume (V_total_) and the volume of micropores (V_micro_ < 2 nm).

The surface functional groups of tested photocatalysts were determined using DRIFT analysis. Spectra were recorded with FT/IR-4200 spectrometer (JASCO International Co., Ltd., Tokyo, Japan) equipped with a DiffuseIR accessory (PIKE Technologies, Fitchburg, WI, USA). Light absorption properties of the samples were studied using UV–Vis spectroscopy. The diffuse reflection absorption spectra at 200-800 nm were recorded on a V-650 spectrophotometer (JASCO International Co., Tokyo, Japan) equipped with an integrating sphere accessory PIV-756 (JASCO International Co., Tokyo, Japan). Barium sulphate (BaSO_4_, pure p.a., Avantor Performance Materials Poland S.A., Gliwice, Poland) was used as the standard in the absorption spectroscopic experiments. The band-gap energy was studied by combining Tauc’s relationship and the Kubelka–Munk function. The method in detail is described in the literature [74].

### 3.3. Photocatalytic Process

The experiments were conducted in the gas phase in a reactor with a volume of 766 cm^3^. The processes utilized a medium-pressure mercury lamp TQ150 Z3 (Heraeus, Germany) with a power of 150 W, characterized by a broad range of radiation in both UV and visible light (250–600 nm, maximum at 365 nm). The lamp was placed in a quartz cooler and constantly supplied with water at 18 °C (Minichiller 280 OLÉ, Offenburg, Germany). The apparatus set was enclosed in a thermostat chamber to eliminate other light sources and maintain a stable process temperature of 20 °C.

For each experiment, the photocatalyst was applied to a glass fiber cut in strips (FF 45 VLIES 50; 40 g/m^2^). The reactor was then filled with 10 cm^3^ of distilled water, and the photocatalyst was placed in it. Subsequently, the reactor’s interior was purged with pure CO_2_ (Messer, Chorzów, Poland) for 16 h. After this time, the system was sealed, and the lamp was turned on. During the process, the gas was mixed using a pump with a flow rate of 1.6 dm^3^/h. The process was conducted for 6 h, and samples for analysis were collected every 1 h. The reactor scheme used in a photocatalytic process has been presented in [75]. The composition of the gas phase after the process was analyzed with a Master GC gas chromatograph (DANI Instruments S.p.A., Milan, Italy) equipped with a 4 m Shincarbon ST 100/120 micropacked column. The detectors used were TCD and FID with a methanizer. The carrier gas was argon. The gas pressure on the column was 6 bars. The volume of the tested gas sample was 1 cm^3^. The content of individual components in the gas phase in subsequent measurements was calculated based on the calibration curve.

### 3.4. Selectivity of the Obtained Materials in the Carbon Dioxide Photoreduction Process

Based on the obtained results, selectivity was calculated and defined as the total ratio between an amount of reactant used to form a certain product per the total amount of this reactant consumed during the process [76], which can be expressed using a formula:

$$S_{A}\left[\%\right]=\frac{n_{A}}{n_{S0}-n_{Se}}\cdot 100\%$$

where:

*S_A_*—selectivity of the substance conversion towards the product *A*,

*n_A_*—the amount of the product *A* moles measured at the end of the process,

*n_So_*—the amount of the substrate moles before the process,

*n_Se_*—the amount of the substrate moles after the process.

This formula has been used to calculate the selectivity values of carbon dioxide conversion process.

Since the H_2_ molecules may be also considered a product of the overall photocatalytic process (due to the parallel water-splitting process), the selectivity of the process towards all products measured in the gas phase (H_2_, CO, and CH_4_) was additionally calculated concerning the total H_2_ present and/or spent during alternative pathways of reaction. To approximate how many moles of H_2_ have been used in the formation of CO or CH_4_ (conversion of CO_2_), (2H+ + 2e^−^) was considered as a molar equivalent of a H_2_ molecule, and allowed to quantify the total amount of H_2_ produced and used during the process.

## 4. Conclusions

In this work, the influence of pH and heat treatment on the physicochemical properties of titania-based materials during photoreduction of carbon dioxide have been studied. Titanium dioxide was synthesized via hydrolysis of titanium (IV) isopropoxide using a sol–gel method, under neutral or basic pH conditions, and heated in the microwave reactor or/and in the high-temperature furnace. Generally, the materials obtained under neutral conditions indicated higher photoactivity than those prepared under basic conditions, probably due to smaller crystallite size of anatase phase. Higher content of hydrogen was detected in the processes using double-heated photocatalysts and photocatalysts heated in the microwave reactor or tubular furnace, prepared in the neutral environment. This shows that the microwave treatment alone is sufficient to synthesize materials with about the same photoactivity as materials that were heated to 400 °C in the tubular furnace. For the materials obtained in alkaline conditions, only double-heated materials and materials heated using microwaves demonstrated higher photoactivity towards the production of hydrogen. Higher contents of methane and carbon monoxide were detected only using double-heated material. For other photocatalysts, the content of these compounds in the gas phase was at a similar level and significant differences were not observed. Higher porosity of the sample positively affected its selectivity of CO_2_ conversion towards methane.

## Figures and Tables

**Table 1:** Average crystallite size, strain, and lattice parameter obtained for studied materials. D_S_—crystallite size calculated using Scherer’s formula, D_W-H_—crystallite size calculated by the Williamson–Hall method, Σ_W-H_—strain calculated by the Williamson–Hall method.

	D_s_	D_W-H_	Σ_W-H_	Lattice Parameter	
	[nm]	[nm]		a = b [Å]	c [Å]
Neutral conditions (without ammonia water)					
T-TiO_2_	5.1	4.9	0.0021	3.795	9.487
T-TiO_2_-FHT-400	8.8	6.0	0.0022	3.794	9.478
T-TiO_2_-FHT-250	6.8	6.5	0.0010	3.793	9.475
T-TiO_2_-MW	6.3	6.8	0.0026	3.791	9.485
T-TiO_2_-MW-FHT	9.0	9.6	0.0042	3.788	9.493
Basic conditions (with ammonia water)					
T-TiO_2_-NH_3_	-	-	-	-	-
T-TiO_2_-NH_3_-FHT-400	21.9	16.0	0.0022	3.781	9.489
T-TiO_2_-NH_3_-FHT-250	-	-	-	-	-
T-TiO_2_-NH_3_-MW	14.3	16.0	0.0021	3.785	9.489
T-TiO_2_-NH_3_-MW-FHT	13.5	16.2	0.0015	3.782	9.488

**Table 2:** Textural properties of the obtained materials.

	S_BET_ [m^2^/g]	TPV [cm^3^/g]	V_micro_ [cm^3^/g]	V_meso_ [cm^3^/g]
Neutral conditions (without ammonia water)				
T-TiO_2_	281	0.30	0.04	0.26
T-TiO_2_-FHT-400	117	0.22	0.01	0.21
T-TiO_2_-FHT-250	199	0.29	0.03	0.26
T-TiO_2_-MW	220	0.34	0.02	0.32
T-TiO_2_-MW-FHT	131	0.27	0.01	0.26
Basic conditions (with ammonia water)				
T-TiO_2_-NH_3_	342	0.27	0.11	0.16
T-TiO_2_-NH_3_-FHT-400	16	0.05	0.00	0.05
T-TiO_2_-NH_3_-FHT-250	163	0.19	0.01	0.18
T-TiO_2_-NH_3_-MW	98	0.28	0.01	0.27
T-TiO_2_-NH_3_-MW-FHT	99	0.25	0.00	0.25

**Table 3:** Calculated selectivity of the photocatalytic process for the obtained samples.

	Selectivity [%]				
	of CO_2_ Conversion		Relative to the Total Amount of H_2_ in the Gas Phase		
Material	CO	CH_4_	H_2_	CO	CH_4_
Neutral conditions (without ammonia water)					
T-TiO_2_	70.3	29.7	53.6	17.3	29.1
T-TiO_2_-FHT-400	80.0	20.0	65.8	17.1	17.1
T-TiO_2_-FHT-250	83.2	16.8	50.8	27.2	22.0
T-TiO_2_-MW	68.2	31.8	68.1	11.1	20.8
T-TiO_2_-MW-FHT-400	78.9	21.1	45.9	26.1	28.0
Basic conditions (with ammonia water)					
T-TiO_2_-NH_3_	91.2	8.8	34.1	47.5	18.4
T-TiO_2_-NH_3_-FHT-400	92.8	7.2	28.2	54.9	16.9
T-TiO_2_-NH_3_-FHT-250	88.5	11.5	34.7	42.9	22.4
T-TiO_2_-NH_3_-MW	82.6	17.4	59.6	21.9	18.5
T-TiO_2_-NH_3_-MW-FHT	84.4	15.6	44.0	32.2	23.8

**Table 4:** Experimental conditions for titanium dioxide heat treatment.

	Heat Treatment	Temperature [°C]
Neutral conditions (without ammonia water)		
T-TiO_2_	-	-
T-TiO_2_-FHT-400	High-temperature furnace	400
T-TiO_2_-FHT-250	High-temperature furnace	250
T-TiO_2_-MW	Microwave reactor	~250
T-TiO_2_-MW-FHT	Microwave reactor; high-temperature furnace	~250, 400
Basic conditions (with ammonia water)		
T-TiO_2_-NH_3_	-	-
T-TiO_2_-NH_3_-FHT-400	High-temperature furnace	400
T-TiO_2_-NH_3_-FHT-250	High-temperature furnace	250
T-TiO_2_-NH_3_-MW	Microwave reactor	~250
T-TiO_2_-NH_3_-MW-FHT	Microwave reactor; high-temperature furnace	~250, 400

## Data Availability

The original contributions presented in the study are included in the article, further inquiries can be directed to the corresponding authors.

## References

[1] Pörtner H.-O., Roberts D.C., Tignor M., Poloczanska E.S., Mintenbeck K., Alegría A., Craig M., Langsdorf S., Löschke S., Möller V. (2022). IPCC Climate Change 2022: Impacts, Adaptation and Vulnerability. Contribution of Working Group II to the Sixth Assessment Report of the Intergovernmental Panel on Climate Change.

[2] Safaei-Farouji M., Misch D., Sachsenhofer F.R. (2023). A review of influencing factors and study methods of carbon capture and storage (CCS) potential in coals. Int. J. Coal Geol..

[3] Ekemezie I.O., Digitemie N.W. (2024). Carbon capture and utilization (CCU): A review of emerging applications and challenges. Eng. Sci. Technol. J..

[4] Al-Enizi M.A., Karim A., Yousef A. (2023). A novel method for fabrication of electrospun cadmium sulfide nanoparticles- decorated zinc oxide nanofibers as effective photocatalyst for water photosplitting. Alex. Eng. J..

[5] Dalal C., Garg A.K., Jain N., Naziruddin A.R., Prajapati R.K., Choudhary S.K., Sonkar S.K. (2023). Sunlight-assisted photocatalytic degradation of azo-dye using zinc-sulfide embedded reduced graphene oxide. Sol. Energy.

[6] Rayappan E., Pandi M., Muthaian J.R., Rosenkranz A., Raji A., Manoj D. (2023). Superior photocatalytic and antibacterial activity of cadmium selenide nanoparticles doped by titanium or lanthanum. Adv. Eng. Mater..

[7] Sandali Y., Butt F.K., Farooq M.H. (2024). Zinc vanadium Oxide/g-C_3_N_4_ nanosheets as potential material for ascorbic acid sensing and photocatalytic reduction of CO_2_. Int. J. Hydrog. Energy.

[8] Annam Renita A., Sathish S., Senthil Kumar P., Prabu D., Manikandan N., Mohamed Iqbal A., Rajesh G., Rangasamy G. (2023). Emerging aspects of metal ions-doped zinc oxide photocatalysts in degradation of organic dyes and pharmaceutical pollutants—A review. J. Environ. Manag..

[9] Zhang Q., Xie G., Duan M., Liu Y., Cai Y., Xu M., Zhao K., Tai H., Jiang Y., Su Y. (2023). Zinc Oxide Nanorods for Light-Activated Gas Sensing and Photocatalytic Applications. ACS Appl. Nano Mater..

[10] Thuan V.D., Ngo L.H., Thi P.H., Chu T.T.H. (2023). Photodegradation of hazardous organic pollutants using titanium oxides—based photocatalytic: A review. Environ. Res..

[11] Xu P., Ding C., Li Z., Yu R., Cui H., Gao S. (2023). Photocatalytic degradation of air pollutant by modified nano titanium oxide (TiO_2_) in a fluidized bed photoreactor: Optimizing and kinetic modeling. Chemosphere.

[12] Liang S., Huang L., Gao Y., Wang Q., Liu B. (2021). Electrochemical Reduction of CO_2_ to CO over Transition Metal/N-Doped Carbon Catalysts: The Active Sites and Reaction Mechanism. Adv. Sci..

[13] Monteiro M.C.O., Philips M.F., Schouten K.J.P., Koper M.T.M. (2021). Efficiency and selectivity of CO_2_ reduction to CO on gold gas diffusion electrodes in acidic media. Nat. Commun..

[14] Wang J., Ji G., Liu Y., Gondal M., Chang X. (2014). Cu_2_O/TiO_2_ heterostructure nanotube arrays prepared by an electrodeposition method exhibiting enhanced photocatalytic activity for CO_2_ reduction to methanol. Catal. Commun..

[15] Graciani J., Mudiyanselage K., Xu F., Baber A.E., Evans J., Senanayake S.D., Stacchiola D.J., Liu P., Hrbek J., Sanz J.F. (2014). Highly active copper-ceria and copper-ceria-titania catalysts for methanol synthesis from CO_2_. Science.

[16] Reñones P., Moya A., Fresno F., Collado L., Vilatela J.J., de la Peña O’Shea V.A. (2016). Hierarchical TiO_2_ nanofibres as photocatalyst for CO_2_ reduction: Influence of morphology and phase composition on catalytic activity. J. CO2 Util..

[17] Morawski A.W., Ćmielewska K., Witkowski K., Kusiak-Nejman E., Pełech I., Staciwa P., Ekiert E., Sibera D., Wanag A., Gano M. (2022). CO_2_ Reduction to Valuable Chemicals on TiO_2_-Carbon Photocatalysts Deposited on Silica Cloth. Catalysts.

[18] Rehman Z.U., Bilal M., Hou J., Butt F.K., Ahmad J., Ali S., Hussain A. (2022). Photocatalytic CO_2_ Reduction Using TiO_2_-Based Photocatalysts and TiO_2_ Z-Scheme Heterojunction Composites: A Review. Molecules.

[19] Tan S.S., Zou L., Hu E. (2006). Photocatalytic reduction of carbon dioxide into gaseous hydrocarbon using TiO_2_ pellets. Catal. Today.

[20] Galli F., Compagnoni M., Vitali D., Pirola C., Bianchi C.L., Villa A., Prati L., Rossetti I. (2017). CO_2_ photoreduction at high pressure to both gas and liquid products over titanium dioxide. Appl. Catal. B Environ..

[21] Garay-Rodríguez L.F., Torres-Martínez L.M. (2020). Photocatalytic CO_2_ reduction over A_2_Ti_6_O_13_ (A = Na and K) titanates synthesized by different pH-catalyzed sol–gel. J. Sol-Gel Sci. Technol..

[22] Yuan Z., Zhu X., Gao X., An C., Wang Z., Zuo C., Dionysiou D.D., He H., Jiang Z. (2023). Enhancing photocatalytic CO_2_ reduction with TiO_2_-based materials: Strategies, mechanisms, challenges, and perspectives. Env. Sci. Ecotechnol..

[23] Ozin G. (2022). Accelerated optochemical engineering solutions to CO2 photocatalysis for a sustainable future. Matter.

[24] Aboualigaledari N., Rahmani M. (2021). A review on the synthesis of the TiO_2_-based photocatalyst for the environmental purification. J. Compos. Compd..

[25] Wang Y., He Y., Lai Q., Fan M. (2014). Review of the progress in preparing nano TiO_2_: An important environmental engineering material. J. Environ. Sci..

[26] Ullattil S.G., Periyat P., Pillai S., Hehir S. (2017). Sol-Gel Synthesis of Titanium Dioxide. Sol-Gel Materials for Energy, Environment and Electronic Applications. Advances in Sol-Gel Derived Materials and Technologies.

[27] Schubert U. (2005). Chemical modification of titanium alkoxides for sol–gel processing. J. Mater. Chem..

[28] Periyat P., Baiju K.V., Mukundan P., Pillai P.K., Warrier K.G.K. (2007). Aqueous colloidal sol–gel route to synthesize nanosized ceria-doped titania having high surface area and increased anatase phase stability. J. Sol-Gel Sci. Technol..

[29] Baiju K.V., Periyat P., Wunderlich W., Pillai P.K., Mukundan P., Warrier K.G.K. (2007). Enhanced photoactivity of neodymium doped mesoporous titania synthesized through aqueous sol–gel method. J. Sol-Gel Sci. Technol..

[30] Kirupavasam E.K., Raj G.A.G. (2013). Preparation, Characterization and Photocatalytic Behaviour of Codoped Nanophotocatalyst. Chem. Sci. Trans..

[31] Milea C., Bogatu C., Duta A. (2011). The influence of parameters in silica sol-gel process. Bull. Transilv. Univ. Bras..

[32] Ren G., Gao Y., Liu X., Xing A., Liu H., Yin J. (2010). Synthesis of high-activity F-doped TiO_2_ photocatalyst via a simple one-step hydrothermal process. Reac. Kinet. Mech. Cat..

[33] Gomathi Thanga Keerthana B., Solaiyammal T., Muniyappan S., Murugakoothan P. (2018). Hydrothermal synthesis and characterization of TiO_2_ nanostructures prepared using different solvents. Mater. Lett..

[34] Mamaghani A.H., Haghighat F., Lee C.-S. (2019). Hydrothermal/solvothermal synthesis and treatment of TiO_2_ for photocatalytic degradation of air pollutants: Preparation, characterization, properties, and performance. Chemosphere.

[35] Akram M., Hussein R., Butt F.K., Latif M. (2019). Study of the effect of microwave holdingtime on the physicochemical properties of titanium oxide. Mater. Res. Express..

[36] Rahimi N., Pax R.A., Gray E.M.A. (2016). Review of functional titanium oxides. I: TiO_2_ and its modifications. Prog. Solid State Chem..

[37] Imoisili P.E., Jen T., Safaei B. (2021). Microwave-assisted sol–gel synthesis of TiO_2_-mixed metal oxide nanocatalyst for degradation of organic pollutant. Nanotechnol. Rev..

[38] Andrade-Guel M., Díaz-Jiménez L., Cortés-Hernández D., Cabello-Alvarado C., Ávila-Orta C., Bartolo-Pérez P., Gamero-Melo P. (2019). Microwave assisted sol–gel synthesis of titanium dioxide using hydrochloric and acetic acid as catalysts. Boletín Soc. Española Cerámica Vidr..

[39] Vlăduț C.M., Mocioiu O.-C., Preda S., Pandele-Cusu J., Bratan V., Trusca R., Zaharescu M. (2022). Effect of Thermal Treatment on the Structure and Morphology of Vanadium Doped ZnO Nanostructures Obtained by Microwave Assisted Sol–Gel Method. Gels.

[40] Divya G., Genji J., Siva Rao T., Chippada M.V., Divyja Lakshmi K.V., Sai Supryja S. (2022). Improved catalytic efficiency by N-doped TiO_2_ via sol gel under microwave irradiation: Dual applications in degrada-tion of dye and microbes. Hybrid Adv..

[41] Predoană L., Petcu G., Preda S., Pandele-Cușu J., Petrescu S.V., Băran A., Apostol N.G., Costescu R.M., Surdu V.-A., Vasile B.Ş. (2023). Copper-/Zinc-Doped TiO_2_ Nanopowders Synthesized by Microwave-Assisted Sol–Gel Method. Gels.

[42] May-Masnou A., Soler L., Torras M., Salles P., Llorca J., Roig A. (2018). Fast and simple microwave synthesis of TiO_2_/Au nanoparticles for gas-phase photocatalytic hydrogen generation. Front. Chem..

[43] Li M., Wang M., Zhu L., Li Y., Yan Z., Shen Z., Cao X. (2018). Facile microwave assisted synthesis of N-rich carbon quantum dots/dual-phase TiO_2_ heterostructured nanocomposites with high activity in CO_2_ photoreduction. Appl. Catal. B Environ..

[44] Yang Z.-Z., He L.-N., Zhao Y.-N., Li B., Yu B. (2011). CO2 capture and activation by superbase/polyethylene glycol and its subsequent conversion. Energy Environ. Sci..

[45] Liu A.-H., Ma R., Song C., Yang Z.Z., Yu A., Cai Y., He L.-N., Zhao Y.-N., Yu B., Song Q.-Q. (2012). Equimolar CO_2_ Capture by N-Substituted Amino Acid Salts and Subsequent Conversion. Ang. Chem. Int. Ed..

[46] Kothandaraman J., Goeppert A., Czaun M., Olah G.A., Surya Prakash G.K. (2016). CO_2_ capture by amines in aqueous media and its subsequent conversion to formate with reusable ruthenium and iron catalysts. Green Chem..

[47] Kar S., Goeppert A., Surya Prakash G.K. (2019). Integrated CO_2_ Capture and Conversion to Formate and Methanol: Connecting Two Threads. Acc. Chem. Res..

[48] Saravanan S., Balamurugan M., Soga T. (2018). Synthesis of titanium dioxide nanoparticles with desired ratio of anatase and rutile phases and the effect of high temperature annealing. Trans. Mater. Res. Soc. Jpn..

[49] Schossberger F. (1942). Z. Kristallogr., Kristallgeom., Kristallphys., Kristallchem..

[50] Meagher E.P., Lager G.A. (1979). Polyhedral thermal expansion in the TiO_2_ polymorphs; refinement of the crystal structures of rutile and brookite at high temperature. Can. Mineral..

[51] Mohamed I.M.A., Dao V.-D., Barakat N.A.M., Yasin A.S., Yousef A., Choi H.-S. (2016). Efficiency enhancement of dye-sensitized solar cells by use of ZrO_2_-doped TiO_2_ nanofibers photoanode. J. Colloid Interface Sci..

[52] Kibasomba P.M., Dhlamini S., Maaza M., Liu C.-P., Rashad M.M., Rayan D.A., Mwakikunga B.W. (2018). Strain and grain size of TiO_2_ nanoparticles from TEM, Raman spectroscopy and XRD: The revisiting of the Williamson-Hall plot method. Results Phys..

[53] Lin Y.-H., Weng C.-H., Srivastav A.L., Lin Y.-T., Tzeng J.-H. (2015). Facile synthesis and characterization of N-doped TiO_2_ photocatalyst and its visible-light activity for photo-oxidation of ethylene. J. Nanomater..

[54] Winter M., Hamal D., Yang X., Kwen H., Jones D., Rajagopalan S., Klabunde K.J. (2009). Defining reactivity of solid sorbents: What is the most appropriate metric?. Chem. Mater..

[55] Abdullah S.M.A., Chong F.K. (2010). Dual-effects of adsorption and photodegradation of methylene blue by tungsten-loaded titanium dioxide. Chem. Eng. J..

[56] Praveen P., Viruthagiri G., Mugundan S., Shanmugam N. (2014). Structural, optical and morphological analyses of pristine titanium di-oxide nanoparticles—Synthesized via sol–gel route. Spectrochim. Acta Part A Mol. Biomol. Spectrosc..

[57] Liu Z., Jian Z., Fang J., Xu X., Zhu X., Wu S. (2012). Low-temperature reverse microemulsion synthesis, characterization, and photocatalytic performance of nanocrystalline titanium dioxide. Int. J. Photoenergy.

[58] Choina J., Dolat D., Kusiak-Nejman E., Janus M., Morawski A.W. (2009). TiO_2_ modified by ammonia as a long lifetime photocatalyst for dyes decomposition. Pol. J. Chem. Technol..

[59] Tryba B., Tygielska M., Colbeau-Justin C., Kusiak-Nejman E., Kapica-Kozar J., Wróbel R., Żołnierkiewicz G., Guskos N. (2016). Influence of pH of sol-gel solution on phase composition and photocatalytic activity of TiO_2_ under UV and visible light. Mater. Res. Bull..

[60] Zhu L., Zhong Z., Yang H., Wang C. (2016). NH_3_-SCR performance of Mn-Fe/TiO_2_ catalysts at low temperature in the absence and presence of water vapor. Water Air Soil Pollut..

[61] Suda Y., Kawasaki H., Ueda T., Ohshima T. (2004). Preparation of high quality nitrogen doped TiO_2_ thin film as a photocatalyst using a pulsed laser deposition method. Thin Solid Films.

[62] Lin Y.-T., Weng C.-H., Hsu H.-J., Lin Y.-H., Shiesh C.-C. (2013). The synergistic effect of nitrogen dopant and calcination temperature on the visible-light-induced photoactivity of N-doped TiO_2_. Int. J. Photoenergy.

[63] Asahi R., Morikawa T., Ohwaki T., Aoki K., Taga Y. (2001). Visible-light photocatalysis in nitrogen-doped titanium oxides. Science.

[64] Amano F., Nogami K., Tanaka M., Ohtani B. (2010). Correlation between Surface Area and Photocatalytic Activity for Acetaldehyde Decomposition over Bismuth Tungstate Particles with a Hierarchical Structure. Langmuir.

[65] Muthuswamy E., Iskandar A.S., Amador M.M., Kauzlarich S.M. (2013). Facile Synthesis of Germanium Nanoparticles with Size Control: Microwave versus Conventional Heating. Chem. Mater..

[66] Wang Z.M., Wang H., Xiao M.Q., Chen X., Dai W., Yu Y., Fu X. (2022). Constructing a channel to regulate the electron transfer behavior of CO adsorption and light-driven CO reduction by H_2_ over CuO-ZnO. ACS Appl. Mater. Interfaces.

[67] Li H., Bharti B., Manikandam V., Al Salhi M.S., Asemi N.N., Wang Y., Jin W., Ouyang F. (2023). Nitrogen-fluorine co-doped TiO_2_/SiO_2_ nanoparticles for the photocatalytic degradation of acrylonitrile: Deactivation and regeneration. Chemosphere.

[68] Haghighi P., Haghighat F. (2024). TiO_2_-based photocatalytic oxidation process for indoor air VOCs removal: A comprehensive review. Build. Environ..

[69] Ricka R., Wanag A., Kusiak-Nejman E., Moszyński D., Filip Edelmannová M., Reli M., Baďura Z., Zoppellaro G., Zbořil R., Morawski A.W. (2024). Photocatalytic reduction of CO_2_ over Ti^3+^ self-doped TiO_2_-based nanomaterials. J. CO2 Util..

[70] Rios R.B., Stragliotto F.M., Peixoto H.R., Torres A.E.B., Bastos-Neto M., Azevedo D.C.S., Cavalcante C.L. (2013). Studies on the Adsorption Behavior of CO_2_-CH_4_ Mixtures Using Activated Carbon. Braz. J. Chem. Eng..

[71] Ismail A.F., Khulbe K., Matsuura T. (2015). Gas Separation Membranes: Polymeric and Inorganic.

[72] Matteucci S., Yampolskii Y., Freeman B.D., Pinnau I. (2006). Transport of Gases and Vapors in Glassy and Rubbery Polymers. Materials Science of Membranes for Gas and Vapor Separation.

[73] Ali S.H., Mohammed S.S., Al-Dokheily M.E., Algharagholy L. (2022). Photocatalytic Activity of Defective TiO_2_-X for Water Treatment/Methyl Orange Dye Degradation. Chem. Chem. Technol..

[74] Jubu P.R., Obaseki O.S., Nathan-Abutu A., Yam F.K., Yusof Y., Ochang M.B. (2022). Dispensability of the conventional Tauc’s plot for accurate bandgap determination from UV–vis optical diffuse reflectance data. Results Opt..

[75] Morawski A.W., Gano M., Ćmielewska K., Kusiak-Nejman E., Pełech I., Staciwa P., Ekiert E., Narkiewicz U. (2023). Photocatalytic reduction efficiency of CO_2_ depending on ZnO particle size. Catalysts.

[76] (2006). IUPAC Compendium of Chemical Terminology.

